# Recombinant *Listeria ivanovii* strain expressing listeriolysin O in place of ivanolysin O might be a potential antigen carrier for vaccine construction

**DOI:** 10.3389/fmicb.2022.962326

**Published:** 2022-07-22

**Authors:** Qian Liang, Ruidan Li, Sijing Liu, Yunwen Zhang, Sicheng Tian, Qian Ou, Zhaobin Chen, Chuan Wang

**Affiliations:** ^1^Department of Public Health Laboratory Sciences, West China School of Public Health and West China Fourth Hospital, Sichuan University, Chengdu, China; ^2^Shen Zhen Biomed Alliance Biotech Group Co., Ltd., Shenzhen, China

**Keywords:** *Listeria monocytogenes*, *Listeria ivanovii*, listeriolysin O, ivanolysin O, immune effect

## Abstract

*Listeria monocytogenes* (LM) induces efficient and specific T-cell immune responses in the host. Listeriolysin O (LLO) is the main virulence protein of LM. LLO helps LM escape from the lysosome. However, the pronounced pathogenicity of LM limits its practical application as a live bacterial vector. *Listeria ivanovii* (LI) also displays intracellular parasitic abilities, cell to cell transfer, and other LM properties, with an elevated biosafety relative to LM. We have confirmed that LI can be used as a viable bacterial vaccine vector. However, we have also observed *in vivo* that LI vector vaccine candidates survive in the immune organ (spleen) for a shorter time compared with the survival time of LM and elicit weaker immune responses compared with LM. Studies have confirmed that hemolysin correlates with some important biological properties of *Listeria*, including cell invasion, intracellular proliferation, and the ability to induce immune responses. We speculated that the weaker immunogenicity of LI compared to LM may be related to the function of ivanolysin O (ILO). Here, we established a hemolysin gene deletion strain, LIΔ*ilo*, and a modified strain, LIΔ*ilo*:*hly*, whose *ilo* was replaced by *hly*. The hemolysin-modified strain was attenuated; however, it led to significantly improved invasive and proliferative activities of antigen-presenting cells, including those of RAW 264.7 macrophages, compared with the effects of LI. Mice immunized twice with LIΔ*ilo*:*hly* showed higher cytokine levels and better challenge protection rates than LI-immunized mice. This is the first description in *Listeria* carrier vaccine research of the modification of LI hemolysin to obtain a better vaccine carrier than LI. The recombinant strain LIΔ*ilo*:*hly* showed good biosafety and immunogenicity, and thus appears to be a good vector strain for vaccine development.

## Introduction

*Listeria monocytogenes* (LM) is a gram-positive food-borne pathogen that is widely distributed in nature. LM can cause listeriosis, which mainly manifests as mild gastroenteritis, but which can be severe and cause meningitis, gastroenteritis, and sepsis (Farber and Peterkin, 1991; Wallecha et al., 2009). LM can proliferate intracellularly. This process is generally considered to be related to listeriolysin O (LLO). LLO is encoded by *hly*, which is located on LM pathogenicity island 1 (LPI-1). LLO is a major virulence factor of LM (Vázquez-Boland et al., 2001). LLO degrades lysosomal membranes to help bacteria escape phagocytic vesicles and enter host cytoplasm for growth. LLO also has an immunomodulatory role and is thus a potential adjuvant for cancer immunotherapy. A previous study showed that fusing LLO protein to human papillomavirus E7 protein improved the tumoricidal function of E7-specific CD8^+^ T cells and increased the number of antigen-specific CD8^+^ T cells in the tumor (Lamikanra et al., 2001), resulting in an improved antitumor therapeutic effect. The unique intracellular lifecycle of LM allows antigens present in or expressed by LM to elicit antigen-specific cellular immune responses through both MHC class I and MHC class II molecular pathways (Harty et al., 1996; Ruan et al., 2016). Therefore, LM is considered to be a promising vector for constructing vaccines that mainly induce cellular immune responses. However, since LM is pathogenic to humans, it must be attenuated before it can be used as a vaccine vector. Many reports have described preventive and therapeutic vaccines that utilize attenuated LM strains as carriers. For example, Yang et al. (2014) described an attenuated LM strain, deleting the *dal* and *dat* genes and carrying the human CD24 gene, led to Hepa1-6-CD24-induced tumor regression and increased tumor-free survival in mice. Tumor therapeutic vaccines based on LM have also shown expected results in clinical trials (Le et al., 2019). Vaccine vectors must have reliable biological safety. However, whether the virulence of LM strains remains attenuated after knocking out virulence or metabolic genes is a major safety issue that remains to be investigated.

*Listeria ivanovii* (LI) was first isolated from a lamb with congenital listeriosis in Bulgaria in 1955. LI rarely infects humans. Thus it displays better biosafety than LM in humans. LI has properties similar to those of LM, such as intracellular growth and cell-to-cell spread, and can enter antigen-presenting cells and proliferate intracellularly (Frehel et al., 2003). We immunized mice with a recombinant LI strain carrying *Mycobacterium tuberculosis* antigen in its genome. We found that the recombinant strain induced antigen-specific CD8^+^ T cell immune responses, confirming that it can be used as a vaccine carrier. However, the immune responses were not very strong (Lin et al., 2015). Ivanolysin O (ILO) differs from LLO in some respects. One study demonstrated that the recombinant strain LIΔ*ilo*:*hly*, constructed by replacing *hly* with ILO coding gene *ilo*, proliferated in mouse livers, but not spleens (Zhou et al., 2016). Since LLO is important for intracellular parasitism and induction of cellular immune responses by LM, we speculated that the weak immunogenicity of LI may be related to the function of ILO. We assumed that the immunogenicity of LI can be improved by replacing its hemolysin with LLO. Improved immunogenicity combined with LI’s increased biosafety may render the modified strain a safe and effective vaccine carrier.

In this study, we constructed a hemolysin gene deletion strain LIΔ*ilo* by knocking out *ilo*. From this strain, we constructed a modified strain, LIΔ*ilo*:*hly* whose *ilo* has been replaced by *hly*. We studied the *in vitro* growth rate, hemolytic titer, and changes in biochemical characteristics of the strain after hemolysin replacement. Next, we studied the adhesion, invasion, and proliferative ability of cells *in vitro*, and the biosafety, immune effect, and immune protection rate *in vivo* of LIΔ*ilo*:*hly*. Replacement of the hemolysin gene did not significantly affect bacterial growth, and LIΔ*ilo*:*hly* maintained biological properties similar to LI. Compared with LI, the *in vitro* proliferation ability of LIΔ*ilo*:*hly* in RAW 264.7 macrophages was significantly improved, but *in vivo* virulence was attenuated; after immunizing animals twice, LIΔ*ilo*:*hly* induced higher cytokine levels and improved the challenge protection rate. The recombinant strain LIΔ*ilo*:*hly* displays good biosafety and immunogenicity, and appears to be a good vector strain for vaccine construction.

## Materials and methods

### Bacteria, plasmids, cells, and animals

Bacteria strains LM 10403s and LI PAM55 were provided by Dr. Hao Shen (Department of Microbiology, Perelman School of Medicine, University of Pennsylvania, Philadelphia, PA, United States), and plasmid pCW-107 was constructed for this study (Wang et al., 2014; Zhou et al., 2016; Mahdy et al., 2019). RAW 264.7, and Hepa1-6 cells were maintained in DMEM (Gibco, New York, NY, United States). Specific-pathogen-free, female C57BL/6 mice (6–8 weeks old) were purchased from Vital River Laboratory Animal Technology Co., Ltd., (Zhejiang, China) and housed at the Animal Center of the School of Public Health in Sichuan University. Animal experiments were approved by the Animal Care and Use Committee of Sichuan University.

### Construction of recombinant strains

Plasmid pCW107 was extracted and digested with *Xba* I and *Not* I restriction enzymes to obtain a linear fragment of the targeting plasmid vector. The LI genome was used as a template to amplify the upstream and downstream homologous sequences of *ilo*. The upstream homologous sequence was inserted into the pCW107 *Xba* I and *Not* I sites to generate plasmid pCW618. This plasmid was digested with *Spe* I and *Not* I, and the downstream homologous sequence was inserted to obtain pCW619. Plasmid pCW619 was cut with *Not* I, then inserted into *lacZ* or *hly* fragments to obtain pCW620 carrying *lacZ* or pCW621 carrying *hly*, respectively. Plasmid pCW620 was electroporated into LI, and strain LIΔ*ilo*:*lacZ* was constructed by homologous recombination (Liu et al., 2020). Plasmids pCW619 and pCW621 were electroporated into LIΔ*ilo*:*lacZ* to construct strains LIΔ*ilo* and LIΔ*ilo*:*hly*, respectively. The strategy used for recombinant bacterial strain construction is shown in Supplementary Figure 1. The primers used are listed in Supplementary Table 1.

### Basic characteristics

#### *In vitro* growth characteristics

Single colonies of LM, LI, LIΔ*ilo*, and LIΔ*ilo*:*hly* were inoculated into 5 mL BHI broth and cultured at 37°C for 18∼24 h. A portion of each culture was inoculated into 40 mL of BHI broth. The inoculum volume that was used resulted in an initial absorbance at 600 nm (*A*_600_) of each resulting suspension of 0.05. Each culture was incubated in a constant-temperature shaker at 200 rpm and at 37°C. Three milliliters were withdrawn every 1 h for 10 h to measure and record the *A*_600_ value. These measurements were used to plot growth curves.

#### Recombinant strain protein expression

LIΔ*ilo*:*hly* was inoculated into BHI broth medium and cultured to the logarithmic phase of growth. The culture supernatant and bacterial precipitate were respectively collected after centrifugation. The total protein from the culture supernatant was extracted by TCA-acetone precipitation, and the total protein from the bacterial precipitate was extracted by ultrasonic breaking and TCA-acetone precipitation. The protein samples were subjected to SDS-PAGE electrophoresis and then transferred onto a PVDF membrane. The membrane was incubated with rabbit anti-listeriolysin O antibody (1:1000) (Abcam, Cambridge, United Kingdom) as the primary antibody, and horseradish peroxidase (HRP)-labeled goat anti-rabbit IgG (H + L) (1:1,000) (Beyotime Biotechnology Co., Ltd., Shanghai, China) as the secondary antibody. The HRP chemiluminescence substrate was applied for color development and the results were analysis after incubation.

#### Bacterial biochemical identification test

Fresh single colonies of LM, LI, LIΔ*ilo*, and LIΔ*ilo*:*hly* were collected and individually added to a tube filled with 4 mL of sterile water. A bacterial suspension was prepared with a McFarland turbidity ranging from 0.4 to 0.6. A bacterial biochemical identification reagent card was placed in the bacterial suspension to sample the bacteria. After removing the bacterial liquid, the reagent card was placed in an automatic microbial identification and drug susceptibility analyzer (Merier Biotechnology Co., Lyon, France) to culture and biochemically identify bacteria.

#### Determination of hemolytic titer

Five milliliters of each logarithmic phase bacterial culture was centrifuged at 13,000 rpm for 5 min. Supernatants were pipetted after centrifugation into wells of a U-shaped 96-well plate and diluted two-fold to 2^–8^. PBS was used instead of culture supernatants to prepare the same dilution for use as a negative control. Thirty microliters of 1% sheep erythrocyte suspension was added to both sample and control wells, and then mixed, and incubated at 37°C for 2 h. The hemolytic titer of each strain was recorded.

### Intracellular proliferation, adhesion and invasion to the cells

#### Proliferation in RAW 264.7 cells

RAW264.7 macrophages were seeded in wells of a 24-well plate at a density of 1 × 10^6^ cells/well and cultured to 70–80% confluency. Next, 100 μL of LM, LI, LIΔ*ilo*, or LIΔ*ilo*:*hly* bacterial suspensions were added to each well at a multiplicity of infection (MOI) of 20:1 and incubated at 37°C for 1 h. Culture medium was discarded, cells were washed with PBS, 1 mL of DMEM medium containing 200 μg/mL gentamicin and 10% fetal bovine serum was added to each well, and cells were cultured at 37°C for 1 h to kill extracellular bacteria. Culture medium was then discarded, cells were washed with PBS, and 0.1% Triton X-100 was added to 2 wells to lyse the cells. After a 1:10 dilution, 20 μL of the suspension was dropped onto the surface of a BHI plate and cultured at 37°C for 48 h. The colony forming unit (CFU) was defined as the total number of bacteria that invaded the cells. The remaining wells were added to DMEM containing 100 μg/mL gentamicin and 10% fetal bovine serum, and incubation was continued. Cells were lysed 2, 4, and 6 h later, and the number of viable bacteria was counted using the above method. Fold changes = (total number of bacteria at each time point)/(total number of bacteria invading cells at 2 h); Multiplication factor (%) = (total number of bacteria at each time point)/(total number of bacteria initially added).

#### Laser scanning confocal microscopy observation of bacteria growth in RAW 264.7 cells

The pCW-*gfp* plasmid expressing green fluorescent protein was electroporated into LM, LI, and LIΔ*ilo*:*hly* to obtain LM-*gfp*, LI-*gfp*, and LIΔ*ilo*:*hly-*gfp**. RAW264.7 cells were plated on a 24-well plate with slides. After the cells had grown to 70–80% confluency, fresh bacterial suspension was added at an MOI of approximately 10:1. After culturing for 1 h, the culture medium was replaced with DMEM containing 30 μg/mL gentamicin. A 24-well plate was removed after 1 and 7 h, and 200 μL immunostaining fixative was added to each well. Cells were fixed at room temperature for 15 min. Supernatants were discarded, and cells were washed with PBS, followed by addition of 200 μL of PBS containing 0.1% Triton X-100 to each well to lyse cell membranes for 10 min at room temperature. After washing, 200 μL PBS-Tween buffer containing 1% BSA and 22.52 mg/ml glycine was added to each well and cells were blocked at room temperature for 30 min. Supernatants were discarded, and pellets were washed. Phagosome staining (Beuzón et al., 2000) was performed using rabbit anti-lysosomal-associated membrane protein 1 (LAMP-1) antibody (Abcam, Cambridge, United Kingdom) and Alexa Fluor 647-conjugated AffiniPure donkey anti-rabbit secondary antibody (Jackson ImmunoResearch, Pennsylvania, United States). After washing, staining solution containing 10 μg/mL 4’,6-diamidino-2-phenylindol (DAPI; Solarbio, Beijing, China) was added to each well in the dark and incubated at room temperature for 5 min. The staining solution was discarded followed by thorough washing, LSCM observation, and photography.

#### Adhesion and invasion rate of the strains to Hepa1-6 cells

Hepa1-6 cells were seeded in 24-well plates at a density of 1 × 10^6^ cells/well and cultured to 70–80% confluency. One hundred microliters of LM, LI, LIΔ*ilo*, or LIΔ*ilo*:*hly* bacterial suspensions were added to each well at an MOI of approximately 20:1.

To determine the adhesion rate, after culturing for 1 h, the 24-well plate was washed with PBS, and 0.1% Triton X-100 solution was added to lyse the cells. Suspensions were then diluted 1:10. Twenty microliters of the diluted suspension was spread onto the surface of a BHI plate and cultured at 37°C for 48 h. CFU was defined as the number of adherent bacteria. The bacterial adhesion rate (%) was calculated as [(number of adherent bacteria)/(total number of bacteria added to cells)] × 100.

To calculate the invasion rate, after culturing for 1 h, the 24-well plate was removed and washed with PBS. DMEM containing 200 μg/mL gentamicin and 10% fetal bovine serum was added to each well and cultured for 1 h. The subsequent operations were the same as described above. CFU is considered to represent the number of invading bacteria. The bacterial invasion rate (%) was calculated as = [(number of invading bacteria)/(total number of bacteria added to cells)] × 100.

### *In vivo* safety and immunogenicity

#### LD_50_ of each strain in C57BL/6 mice

The 50% lethal dose (LD_50_) of LI was previously determined to be 6.3 × 10^6^ CFU per mouse (Lin et al., 2015). To determine LD_50_ for each of the other strains, C57BL/6 mice were injected with 100 μL of the bacterial suspension through the tail vein. The dose groups and inoculation volumes for each strain are shown in Supplementary Table 4. Deaths among the mice were recorded for 10 days. LD_50_ values were determined using an improved Karber method.

#### Determination of bacterial load in organs

C57BL/6 mice were intravenously inoculated with LM, LI, LIΔ*ilo*, or LIΔ*ilo*:*hly* at a dose of 0.1 × LD_50_ of each strain. The mice were observed daily, and their body weights were recorded. Mice were sacrificed 1, 3, 5, 7, 9, and 14 days post-injection. The liver, spleen, and lungs were collected aseptically and homogenized in PBS containing 0.1% Triton X-100. The homogenate (50 μL) was diluted 1:10, and 20 μL was dropped onto the surface of a BHI plate. Plates were incubated at 37°C for 48 h. Colonies were counted to determine CFU values.

#### Determination of serum alanine aminotransferase and aspartate aminotransferase levels

At 1, 3, 5, 7, 9, and 14 days after injection, blood was collected from the mice. The serum was separated to detect the levels of alanine aminotransferase (ALT) and aspartate aminotransferase (AST).

#### Tissue sections and pathological observation

Liver, spleen, and lungs were aseptically dissected on days 3 and 4 after injection. Tissues were perfused with 4% paraformaldehyde solution and sent to Sewell Biotechnology Co., Ltd., (Wuhan, China) for section preparation and observation/pathological grading.

#### Determination of cytokine levels

Mice were randomly divided into four groups. Each mouse was intravenously inoculated twice at an interval of 7 days with LM, LI, or LIΔ*ilo*:*hly* at a dose of 0.1 × LD_50_ of each strain, or with PBS as a blank control. The mice were sacrificed 9 and 40 days after the second immunization. Splenocyte suspensions were added to 24-well plates. Mixed LLO peptides (LLO318-329 AFDAAVSGKSVS; LLO297-304 AYGRQVYL; LLO253-264 QIYYNVNVNEPT; LLO91-99 GYKDGNEYI; LLO190-201 NEKYAQAYPNVS) were used as stimulants to stimulate LM and LIΔ*ilo*:*hly* groups for 48 h. ILO protein was used for the LI group. Plates were centrifuged at 1,200 rpm for 20 min and supernatants were collected. Levels of tumor necrosis factor-alpha (TNF-α), interleukin (IL)-12, IL-6, IL-4, and interferon-gamma (IFN-γ) were determined according to ELISA kit (Elabscience, China) instructions.

#### Determination of immune protection rate

The LM, LI, LIΔ*ilo*, and LIΔ*ilo*:*hly* groups were immunized with the corresponding strains, as described above. Challenges were conducted 7 days after the second immunization with 5 × LD_50_ of LM for the LM group and 5 × LD_50_ of LI for the LI, LIΔ*ilo*, LIΔ*ilo*:*hly*, and PBS groups *via* the tail vein. Mice were observed for 14 days, deaths were recorded, and protection rates were calculated.

### Statistical analysis

Data were analyzed using SPSS 21.0 (IBM, United States). The data with a normal distribution are expressed as means ± standard deviation. One-way ANOVA was used for parametric tests, and the LSD test was used for pairwise comparisons between groups. Non-parametric data were analyzed using the Kruskal-Wallis non-parametric test. Count data were analyzed using Fisher’s probability method. *P* < 0.05 indicated statistical significance.

## Results

### Targeting plasmid and recombinant strain construction

Four recombinant plasmids (pCW618, pCW619, pCW620, and pCW621) were obtained by amplifying target fragments using PCR, followed by enzyme digestion, ligation, and transformation. The *lacZ* gene fragment carried by the pCW620 plasmid was integrated into LI by electrotransformation and homologous recombination. LIΔ*ilo*:*lacZ* was obtained, and pCW619 and pCW621 were electro-transformed into LIΔ*ilo*:*lacZ*. After homologous recombination, blue-spot and white-spot screening, antibiotic screening, genome PCR, and sequencing were conducted to verify the successful construction of LIΔ*ilo* and LIΔ*ilo*:*hly* (Supplementary Figure 2).

### Growth of the strains

**FIGURE 1:**
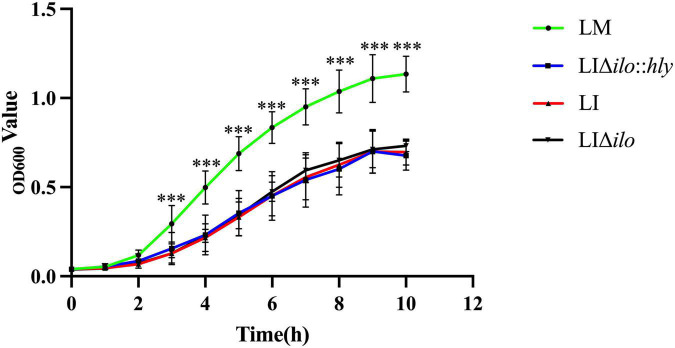
The growth curves of the strains. ^***^P < 0.001 vs. *Listeria monocytogenes* (LM). Experiments were carried out at least three times.

The growth of LM, LI, LIΔ*ilo*, and LIΔ*ilo*:*hly* reached the logarithmic phase after 2 h of culture. Growth plateaued 9 h after inoculation. After 3 h, the growth rate of LM was higher than that of LI, LIΔ*ilo*, and LIΔ*ilo*:*hly*. The growth rate differences between LM and the other three strains were statistically significant. The rate and trend of the growth of LIΔ*ilo*:*hly* and LIΔ*ilo* were consistent with those observed for LI at each time point (Figure 1). The growth difference was not statistically significant, indicating that *ilo* knockout and *hly* complementation did not affect the *in vitro* growth of the recombinant strains.

### Analysis of protein expression of recombinant strain

**FIGURE 2:**
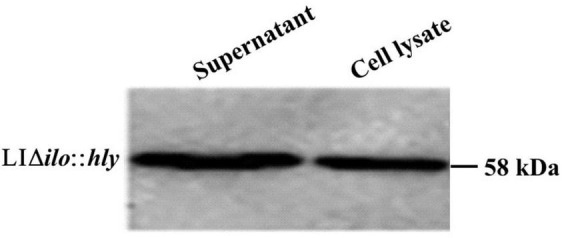
Western blot detection results of hemolysin-modified strain LIΔ*ilo*:*hly.* Supernant: the sample was the protein extracted from 35 mL medium supernatant; Cell lysate: the sample was the protein extracted from the bacterial pellet collected from 6 mL bacteria culture.

The culture supernatant and bacterial pellet of LIΔ*ilo*:*hly* were collected, and the total protein was extracted from culture supernatant and bacterial pellet, respectively. The obtained protein samples were denatured and subjected to SDS-PAGE electrophoresis and western blot. The specific-band with expected length (58 KD) was obviously visible in both the medium supernatant and the cell lysate protein samples, indicating that the recombinant bacteria successfully expressed and secreted the LLO protein (Figure 2).

### Biochemical properties of strains

Most of the biochemical properties of the hemolysin-deletion strain LIΔ*ilo* and hemolysin-modified strain LIΔ*ilo*:*hly* were consistent with those of LI (Supplementary Table 1). The hemolytic titer of LIΔ*ilo*:*hly* was higher than that of LM, but lower than that of LI (Supplementary Figure 3).

### Intracellular proliferation, cell adhesion, invasion, and lysosome escape

**FIGURE 3:**
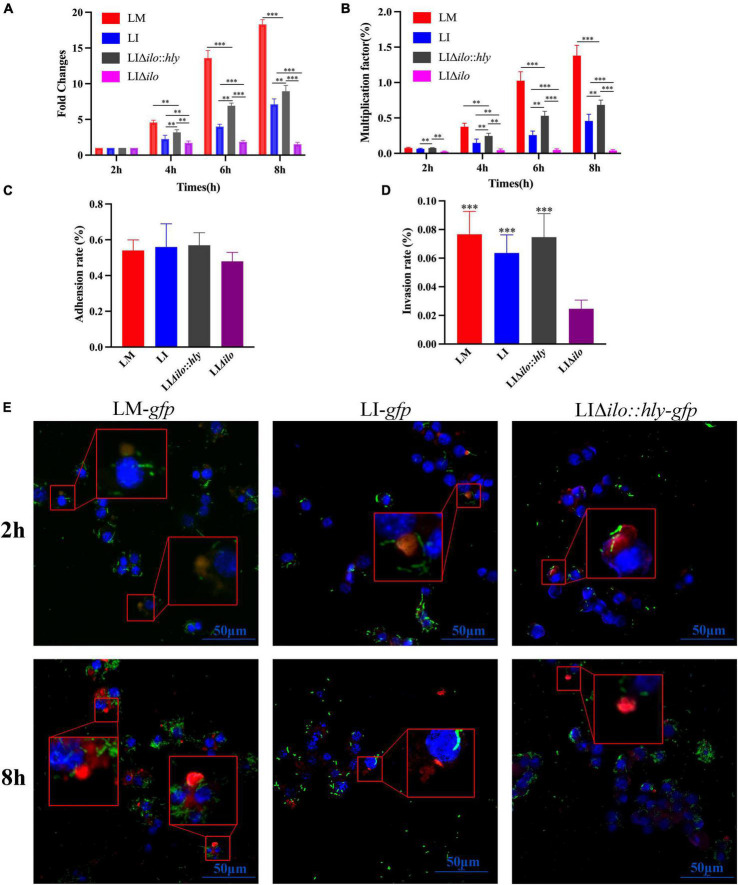
Intracellular proliferation, cell adhesion and invasion and lysosome escape ability. The fold changes **(A)** and multiplication factor **(B)** of each strain in mouse macrophage RAW 264.7, ^**^P < 0.01, ^***^P < 0.001; The adhesion **(C)** and invasion **(D)** ability of each strain to Hepa1-6 cells, the experiments were performed with biological triplicates, and results are expressed as means ± SEM per group, ^***^P < 0.001, vs. LIΔ*ilo*. Experiments were carried out at least three times. The proliferation of each strain at 2 and 8 h post infection in RAW 264.7 observed by Laser scanning confocal microscopy (LSCM) **(E)**, blue, green, and red were labeled the nucleus, bacteria and lysosomes respectively. The lysosomes in Figure 3D were orange or pink at 2 h (bacteria have not escaped), but were red at 8 h (bacteria had escaped). The scale bar in Figure 3D is 50 μm.

RAW264.7 macrophages were infected with LM, LI, LIΔ*ilo*:*hly*, and LIΔ*ilo*, and intracellular bacterial numbers were determined at 2, 4, 6, and 8 h post-infection. All strains except LIΔ*ilo* proliferated in macrophages. LIΔ*ilo*:*hly* showed the same proliferation trend as that of LI. Its intracellular proliferation ability was weaker than that of LM but stronger than that of LI, indicating that the intracellular proliferation ability of the modified strain was improved to a certain extent (Figures 3A,B).

The adhesion rates of LM, LI, LIΔ*ilo*, and LIΔ*ilo*:*hly* to Hepa1-6 cells were 0.54, 0.56, 0.57, and 0.48%, respectively (Figure 3C). Knockout of *ilo* did not affect the ability to adhere to mouse hepatocellular carcinoma cells. The invasion rates of LM, LI, LIΔ*ilo*:*hly*, and LIΔ*ilo* into Hepa1-6 cells were 0.076, 0.062, 0.075, and 0.024%, respectively (Figure 3D). LIΔ*ilo* had the lowest invasion rate, indicating that knockout of *ilo* affected invasiveness, and that replacement with *hly* restored the strain’s invasiveness.

Laser scanning confocal microscopy (LSCM) of the infected macrophages (Figure 3E) showed that the bacterial numbers at 8 h were significantly higher than those at 2 h. LM-*gfp* showed the strongest proliferative ability, followed by LIΔ*ilo*:*hly-*gfp** and LI-*gfp*. At 2 h post-infection, there were more green fluorescent bacteria in the lysosome. At 8 h post-infection, there were almost no bacteria in the lysosome and many bacteria appeared in the cytoplasm, indicating that the bacteria had escaped from the lysosomes. Because the total number of bacteria at 8 h was different between the groups, the escape ability of the strains could not be accurately compared.

### *In vivo* biosafety evaluation

#### Determination of LD_50_, bacterial load in organs, and levels of alanine aminotransferase and aspartate aminotransferase

**FIGURE 4:**
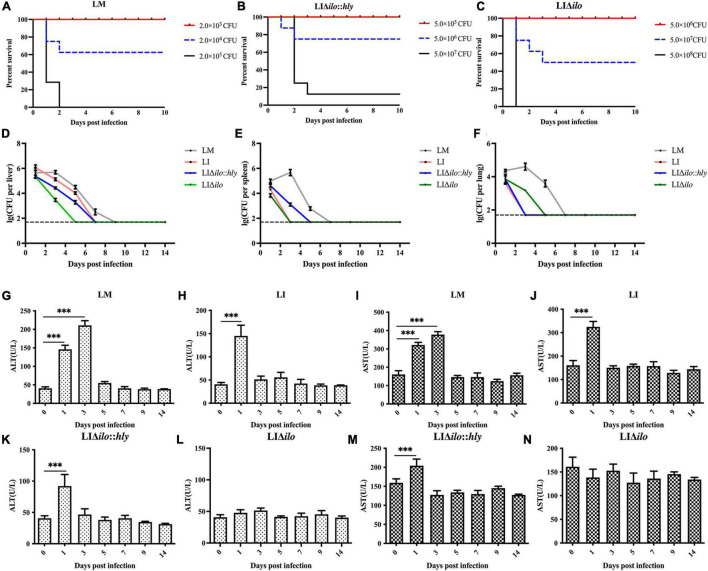
*In vivo* biosafety evaluation of each strain. Survival curves of C57BL/6 mice intravenously inoculated of *Listeria monocytogenes* (LM) **(A)**, LIΔ*ilo*:*hly* **(B)**, and LIΔ*ilo* **(C)**; Eight mice per group in this experiment. The bacteria load in liver **(D)**, spleen **(E)**, and lung **(F)** of mice intravenously inoculated of each strain; each point represents the mean ± SEM for a group of three mice from one independent experiment. Mice serum ALT levels after inoculation of LM **(G)**, LI **(H)**, LIΔ*ilo*:*hly* **(K)**, and LIΔ*ilo* **(L)**, and mice serum AST levels after inoculation of LI **(J)**, LM **(I)**, LIΔ*ilo*:*hly* **(M)**, and LIΔ*ilo* **(N)**. Results are expressed as means ± SEM per group, ^***^P < 0.005. The experiments were performed with biological triplicates.

To evaluate the virulence of each strain *in vivo*, the LD_50_ of LM, LIΔ*ilo*:*hly* and LIΔ*ilo* in C57BL/6 mice was determined. LD_50_ results were 4.3 × 10^4^ CFU/mouse for LM, 1.1 × 10^7^ CFU/mouse for LIΔ*ilo:hly*, and 4.3 × 10^7^ CFU/mouse for LIΔ*ilo*. The LD_50_ of LI was 6.3 × 10^6^ CFU/mouse (determined in our laboratory) (Lin et al., 2015). Virulence *in vivo* was significantly attenuated by *ilo* knockout. Although virulence was restored after *hly* compensation, the strain still had reduced virulence compared with LI, confirming the increased biosafety of LIΔ*ilo*:*hly* (Figures 4A–C).

C57BL/6 mice were inoculated with 0.1 × LD_50_ of each strain as the immunizing dose. The growth of the strains in the main organs of the mice is shown in Figures 4D–F. Bacterial numbers in the liver, spleen, and lung of the LM immunized mice group reached a peak on day 3, while peak bacterial numbers in the liver, spleen, and lung of the LI, LIΔ*ilo*:*hly*, and LIΔ*ilo* immunized mice groups were observed on day 1. Bacterial numbers gradually decreased thereafter, indicating that the body quickly began to clear bacteria. Notably, the mice required two more days to completely remove LIΔ*ilo*:*hly* from the spleen than to clear LI from the spleen.

Serum ALT and AST levels reflect the status of the liver. As shown in Figures 4G–N, the ALT and AST levels in the serum of mice after LIΔ*ilo* inoculation did not change significantly, indicating that LIΔ*ilo* inoculation caused no obvious damage to the liver. At day 1 after inoculation, the levels of ALT and AST in the serum of LM-, LI-, and LIΔ*ilo*:*hly* inoculated mice were significantly higher than those before inoculation (*P* < 0.001). Afterward, they began to decrease and returned to the level of the control group on day 5 in the LM group or on day 3 in the LIΔ*ilo*:*hly* group. This trend is consistent with the above bacterial organ load trend.

### Organ pathological examination

**FIGURE 5:**
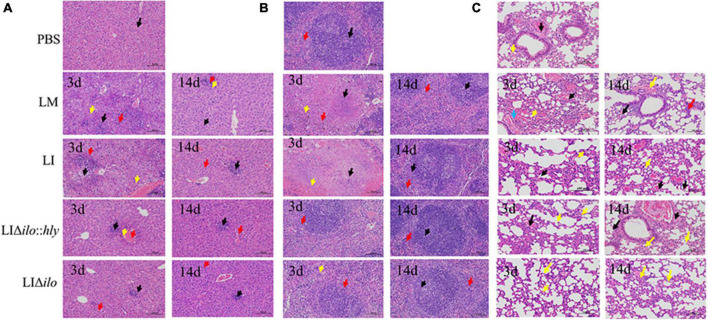
Pathological changes of mouse organs. Pathological changes of Liver **(A)**, spleen **(B)**, and lung **(C)** after inoculation of each strain (200×). The arrows in panel **(A)** indicated the lesions as below: ballooning or cytoplasmic vacuolation (black arrows); small foci of necrosis (yellow arrows), and inflammatory cell infiltration (red arrows); The arrows in panel **(B)** indicated the lesions as below: central expansion of the white pulp or pyknotic fragmentation (black arrows); extensive hemorrhage or multinucleated giant cells (yellow arrows), and inflammatory cell infiltration (red arrows); The arrows in panel **(C)** indicated the lesions as below: minor hemorrhage in the alveolar space (blue arrow); central dilation of the white pulp or pyknosis and fragmentation (black arrow); small hemorrhage (red arrow), and inflammatory cell infiltration (yellow arrow).

After tail vein inoculation, the pathological changes in the liver, spleen, and lung showed a consistent trend in each group. A visible pathological change occurred on day 3, but the pathological status was restored to normal by day 14 (Figures 5A–C). The pathological status of the LIΔ*ilo*:*hly* group at day 3 was better than that of the LM and LI groups. In the liver, a small amount of inflammatory cell focal infiltration in the hepatic lobule (black arrow) was accompanied by inflammatory cell punctate infiltration (red arrow). In the spleen, there was occasional pyknosis and fragmentation accompanied by a small amount of inflammatory cell infiltration. The pathological degree of the spleen was weaker than that of the LM group and the LI group. In the lung, a small amount of alveolar wall around the airway of the lung tissue was slightly thickened (black arrow), the alveolar space was narrow, and inflammatory cell infiltration was scattered. Pathological scores for each organ are shown in Supplementary Table 3. The overall pathological severity of the LIΔ*ilo*:*hly* group was lower than that of the LM and LI groups, indicating that *hly* supplementation improved the biosafety of the strain.

### *In vivo* immunogenicity and immune protection

**FIGURE 6:**
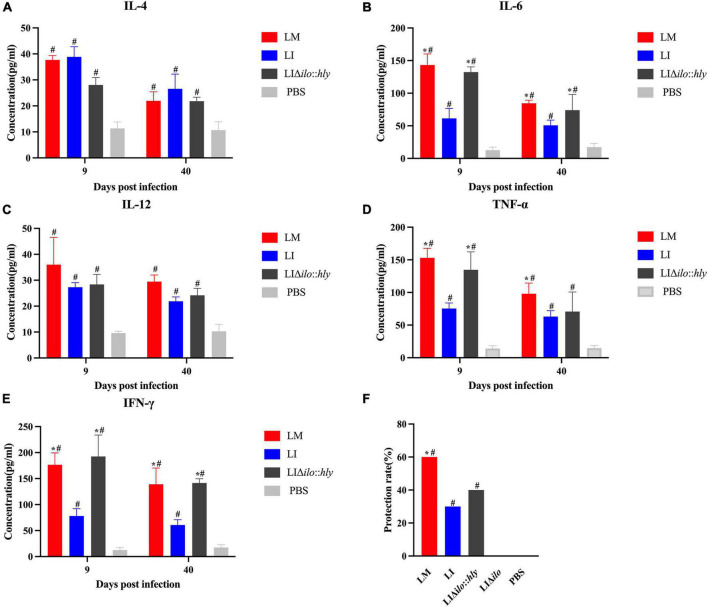
Immunogenicity and immune protection of each strain. Cytokines IL-4 **(A)**, IL-6 **(B)**, IL-12 **(C)**, TNF-α **(D)**, and IFN-γ **(E)** at the 9th and 40th day after the secondary immunization of each strain, *P < 0.05, vs. *Listeria ivanovii* (LI); ^#^*P* < 0.05, vs. PBS, the experiments were performed with biological triplicates. Each bar represents the mean ± SEM for a group of three mice from one independent experiment; Challenge protection rate after twice immunizations **(F)**, *P < 0.05, vs. LI; ^#^*P* < 0.05, vs. PBS. Ten mice per group in this experiment.

The mice in each group were immunized twice by tail vein injection. Spleens were collected aseptically at on days 9 and 40 after the second immunization to prepare splenocyte suspensions for cytokine determination. TNF-α, IL-4, IL-12, IL-6, and IFN-γ levels in the LM, LI, and LIΔ*ilo*:*hly* groups were higher than those in the PBS control group. The levels of TNF-α, IL-6, and IFN-γ at day 9 and the levels of IL-6 and IFN-γ at day 40 in the LIΔ*ilo*:*hly* group were higher than those in the LI group (Figures 6A–E). Mice were challenged 1 week after the second immunization. The protection rates of the LM, LI, LIΔ*ilo*:*hly*, LIΔ*ilo*, and PBS groups were 60, 30, 40, 0, and 0%, respectively. There was a statistically significant difference in the protection rate between LIΔ*ilo*:*hly* and PBS groups. The protection rate of the LIΔ*ilo*:*hly* group was 10% higher than that of the LI group. However, the difference was not significant (Figure 6F).

## Discussion

A qualified live bacterial vaccine carrier should be safe for the host, and should simultaneously stimulate the host immune system to generate a corresponding immune response. Improving the biosafety of LM as a vaccine carrier is an important goal for researchers. Most attenuation strategies adopted for LM are based on inactivating virulence. Although virulence has been greatly reduced, potential biosafety problems remain. Sometimes, deletion of the key virulence genes of *Listeria* will affect its ability to grow and proliferate *in vitro* and *in vivo*, and also affect its ability to stimulate cellular immune responses and the efficiency of antigen presentation (Maciag et al., 2009; Shahabi et al., 2011; Fares et al., 2019). Insufficient attenuation of LM may lead to potential safety problems. Conversely, excess attenuation of LM may lead to low immunogenicity. Therefore, we used a different approach by selecting LI, which displays good biosafety, as the vaccine carrier. To improve the immunogenicity of LI, we replaced the *ilo* gene with *hly*. LLO is an important virulence protein in LM that triggers innate and adaptive immune responses (Mandal and Lee, 2002; Radford et al., 2002). LLO is also utilized as an adjuvant when constructing subunit vaccines to improve the immunogenicity of the antigen. LLO can stimulate the innate immune system and induce the expression of IL-1, IL-12, and IL-18 in macrophages, and the production of IFN-γ by natural killer cells (Kohda, 2002; Carrero et al., 2012). A previous study constructed a recombinant *Escherichia coli* strain expressing LLO and ovalbumin (OVA) and found that it could kill OVA-expressing melanoma cells (B16-OVA) and effectively inhibit tumor growth in mice. Additionally, LLO can mediate bacterial escape from phagosomes and promote bacterial proliferation during infection. LLO plays a crucial role in the control and specific regulation of immune responses. Therefore, in this study, we modified the hemolysin gene of LI with the expectation of full use of the unique advantages of LLO to obtain an excellent vaccine vector.

In this study, *hly* was complemented by *ilo* deletion in the LI strain. The cell invasion and proliferation abilities, biosafety, and immunogenicity of the modified strain LIΔ*ilo*:*hly* were evaluated. The modified strain grew stably *in vitro* and maintained a growth trend similar to that of wild type LI, and it successfully expressed and secreted LLO protein. It could proliferate in phagocytic cells, and its proliferative capacity was stronger than that of LI, confirming that the intracellular proliferative capacity is closely related to hemolysin, and LLO is more helpful in phagocytic vesicle escape and intra-cytoplasm proliferation. Knockout of *ilo* significantly reduced cell invasion ability, and replacement with *hly* restored invasion, indicating that hemolysin is also related to cell invasion. In addition to hemolysin, the internalins of *Listeria* are also closely related to its adhesion and invasion properties. Internalins A (*InlA*) and Internalins B (*InlB*) play their respective roles by binding to specific receptors through leucine rich repeats (LRRs), of which *InlA* mainly binds Cadherin mediates the passage of bacteria across epithelial cells, and *InlB* mainly binds to the hepatocyte growth factor receptor (HGFR) and mediates the entry of bacteria into fibroblasts, hepatocytes and epithelial cells (Vázquez-Boland et al., 2001; Domínguez-Bernal et al., 2006; Hamon et al., 2006). The LD_50_ of LIΔ*ilo*:*hly* was higher than that of LI, and the overall pathological status of the liver, spleen, and lungs was better than that of LI, suggesting that it is less virulent and safer than LI. We speculate that replacement of *ilo* by *hly* may affect the expression levels of virulence genes encoded by *Listeria* pathogenicity islands (LIPI). LIPI is recognized as unstable chromosomal regions, carrying genes related to specific metabolic activities, antibiotic resistance, or pathogenesis, which can be horizontally transferred between bacteria, and contains the essential virulence genes (*prfA*, *hly*, *plcA*, *mpl*, *actA*, and *plcB*) and internalin genes of *Listeria* (Hacker and Kaper, 2000; Vázquez-Boland et al., 2001). We also speculate that the synergy effect of *hly* gene with other virulence genes is weakened within the genome environment of LI. After LIΔ*ilo*:*hly* infection, it was maintained in the liver of mice with a higher bacterial number and a longer duration than in other organs, indicating its superiority in colonizing the liver. This is mainly because the internalin inlB of *Listeria* can bind to specific receptors on the surface of liver cells, resulting in the targeted invasion of the liver by Listeria (Khelef et al., 2006; Pentecost et al., 2010). The clearance time for LIΔ*ilo*:*hly* from the spleen was two more days than that for LI, indicating that LLO has better spleen cell adaptability than ILO. This result also confirmed a previous report that LMΔ*hly*:*ilo* cannot proliferate in the spleen (Frehel et al., 2003). We speculate that the longer survival time of LIΔ*ilo*:*hly* in the spleen may elicit stronger immune responses. After mice were immunized twice with each strain, we detected levels of IFN-γ, TNF-α, IL-4, IL-6, and IL-12. These cytokines coordinate an effective immune response during *Listeria* infection, and are closely related to the establishment of protection. Their interactions promote the proliferation and activation of immune cells, resulting in a stronger and longer-lasting cellular immune response. The results showed that on days 9 and 40 after the secondary immunization of mice with LIΔ*ilo*:*hly*, these cytokines were significantly higher than those in the PBS group, and were comparable to those in the LM group. Especially, the levels of IFN-γ, IL-6, and TNF-α (at day 9) were higher than those of LI group. In addition, the challenge results showed that the immune protection rate of LIΔ*ilo*:*hly* was higher than that of LI. These results are consistent with our hypotheses. A longer survival time in the spleen can result in a more durable, specific immune response. LIΔ*ilo*:*hly* has comparable potency to LM in causing a T helper 1 (Th1) cell-mediated immunity response and inducing various cytokines and chemokines.

The recombinant strain LIΔ*ilo*:*hly* was successfully constructed. Through evaluations at both the cellular and animal levels, we confirmed its safety and immunogenicity. Thus, it can be used as a vaccine carrier. In the future, the heterologous antigen genes can be introduced into the recombinant LI strain by genome recombination technique described previously (Wang et al., 2014) or by recombinant plasmid that carrying heterologous antigen genes, thus to acquire the potential vaccine candidates. Subsequently, it is necessary to explore whether the vaccine candidates can stably express and secret foreign genes by western blot, and through animal experiments to determine antibody titers and measure T/B cell immune responses, to evaluate the safety and efficacy of vaccine candidate strains (Xiao et al., 2021; Clint et al., 2022). Thus to prove that LIΔ*ilo*:*hly* is an excellent antigen carrier for vaccine development, contributing to cancer immunotherapy and infectious diseases prevention.

## Data availability statement

The original contributions presented in this study are included in the article/Supplementary material, further inquiries can be directed to the corresponding authors.

## Ethics statement

The animal study was reviewed and approved by the Animal Care and Use Committee of Sichuan University.

## Author contributions

CW and ZC conceived and designed the research. QL, RL, SL, YZ, ST, and QO performed the experiments and acquired, interpreted, and analyzed the data. QL, RL, SL, and CW wrote the manuscript. All the authors read and critically reviewed the manuscript and approved the submitted version.

## Conflict of interest

QL, RL, and ZC were employed by Shen Zhen Biomed Alliance Biotech Group Co., Ltd. The remaining authors declare that the research was conducted in the absence of any commercial or financial relationships that could be construed as a potential conflict of interest.

## Publisher’s note

All claims expressed in this article are solely those of the authors and do not necessarily represent those of their affiliated organizations, or those of the publisher, the editors and the reviewers. Any product that may be evaluated in this article, or claim that may be made by its manufacturer, is not guaranteed or endorsed by the publisher.
