# Supplementary material for: Recombinant Listeria ivanovii strain expressing listeriolysin O in place of ivanolysin O might be a potential antigen carrier for vaccine construction
Source: Front Microbiol. 2022 Jul 22;13:962326. doi: 10.3389/fmicb.2022.962326 (PMC9355162; doi:10.3389/fmicb.2022.962326)
Supplement: Supplementary file 1 [file Data_Sheet_1.docx]

**Supplementary Materials**

**Figure S1.**


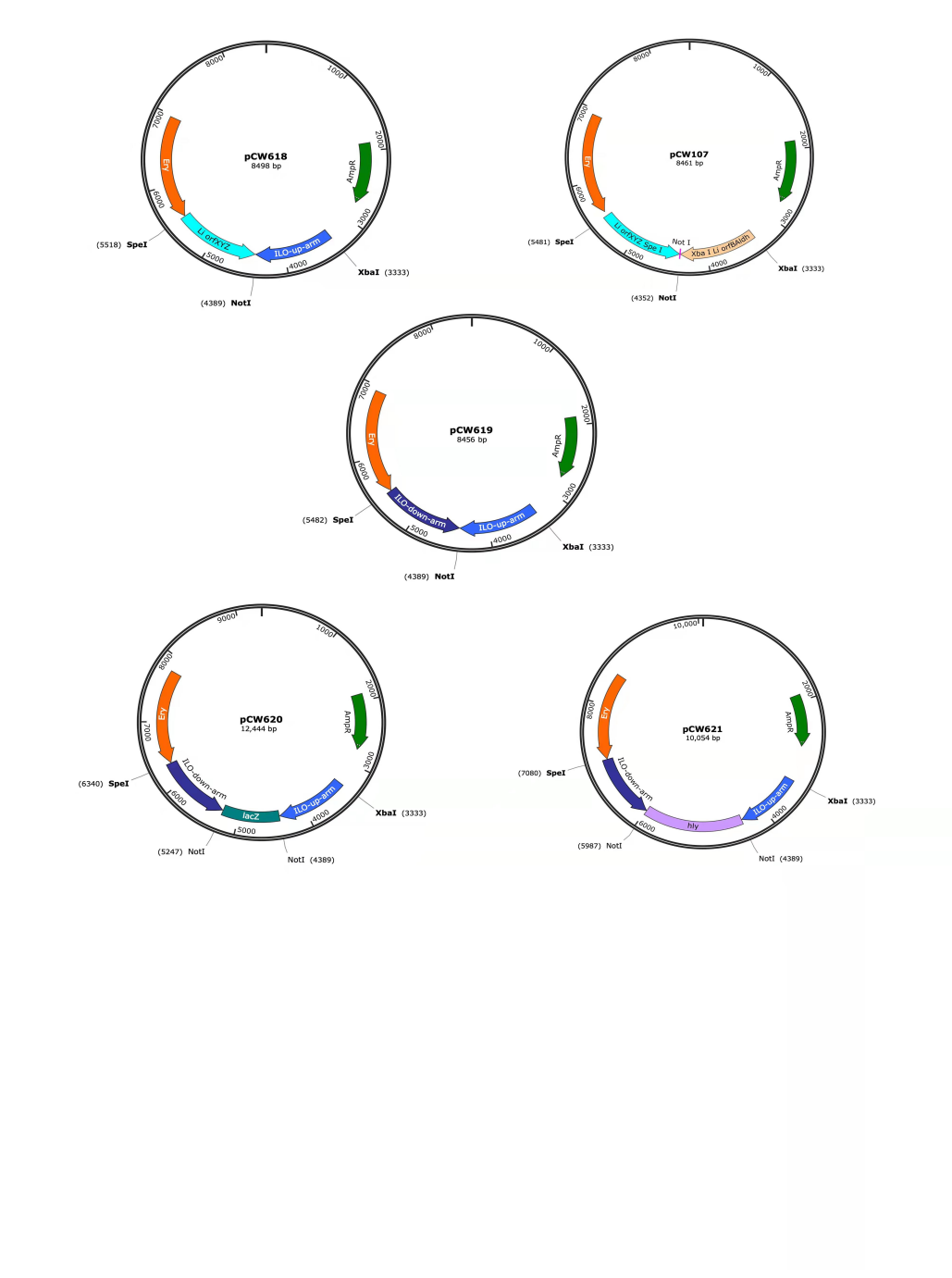

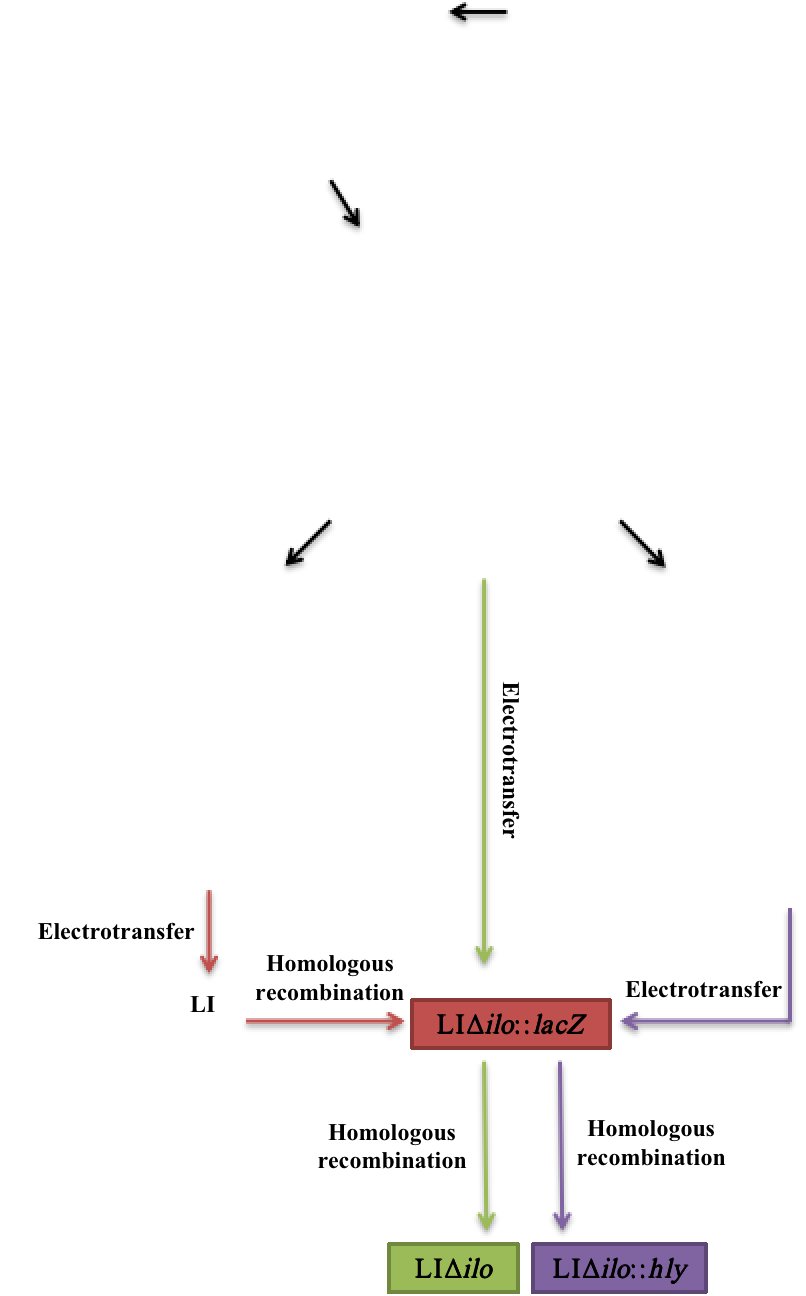


**Plasmid and recombinant bacteria construction flow chart.**

**Figure. S2.**

**
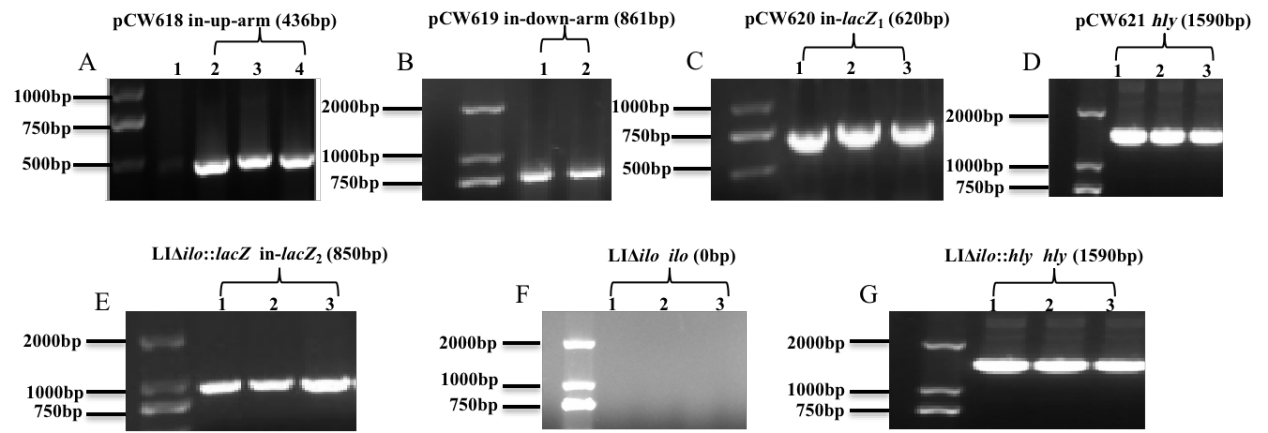
**

**Targeting plasmids and target strains were used to construct related electropherograms.** The up-arm was ligated with the linear vector of pCW617 digested by *XBa*Ⅰand *Not*Ⅰ, and amplified with primers in-up-arm-f and in-up-arm-r (Figure A. lane 2-4); down-arm It was ligated with the linear vector of pCW618 digested by *Spe*Ⅰand *Not*Ⅰ, and amplified with primers in-down-arm-f and in-down-arm-r (Figure B. lane 1-2); *lacZ* and pCW619 were digested with *Not*Ⅰ enzyme The cut linear vector was ligated and amplified with primers in-lacZ_1_-f and in-*lacZ*_1_-r (Figure C. lanes 1-3); *hly* was ligated with the linear vector of pCW619 digested by *Not*Ⅰ, and then the primer in-*lacZ*1-r was used. Amplification results of -*hly*-f and in-*hly*-r (Figure D. lanes 1-3); pCW620 was electrotransformed into LI and amplified with primers in-*lacZ*_2_-f and in-*lacZ*_2_-r after homologous recombination (Figure E. lanes 1-3); pCW619 was electro-transformed into LIΔ*ilo*::*lacZ* and amplified with primers *ilo*-f and *ilo*-r after homologous recombination (Figure F. lanes 1-3); pCW621 was electro-transformed into LIΔ*ilo*::*lacZ* by homologous recombination The results were amplified with primers *hly*-f and *hly*-r after homologous recombination (Figure G. lanes 1-3).

**Figure S3.**


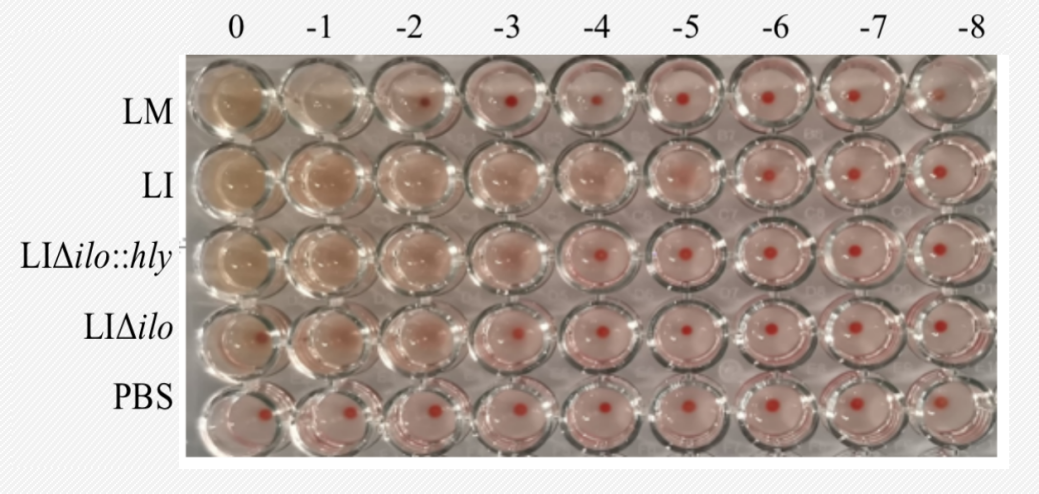


**hemolytic titers of LM, LI, LIΔ*ilo* and LIΔ*ilo*::*hly*.** Hemolytic phenomenon of the strains in a U-shaped 96-well plate**.** The experimental results showed that the hemolytic titers of LM, LI, and LIΔ*ilo*::*hly* were 2^-2^, 2^-6^, and 2^-4^, respectively. The hemolytic titer of LIΔ*ilo*::*hly* was 4 times lower than that of LI, but 4-fold higher than that of LM. *n =* 3.

Table S1.

引物序列及扩增基因长度

| **Primer name** | **Forward primer（5’→3’）** | **Reverse primer（5’→3’）** | **Amplification length（bp）** |
| --- | --- | --- | --- |
| *ilo-*up | GCAGATACACTTAAAAAAGTTCTAGAGCAAGTTAAGGAAGTAGCGC | TTTCCATATTTTTAGCGGCCGCGGTTTTCACTCTCCTTCTAC | 1063 |
| *ilo-*down | AGTGAAAACCGCGGCCGCTAAAAATATGGAAAACCC | AATTCGCCCGGGACTAGTTTATGGTTCAAATCAAACGTT | 1107 |
| in-up*-*arm | TAAGGAAGTCCAAACA | GTCCTCCATACCGTAACTCG | 436 |
| in-up*-*down | CCGATGTGACGGTTAATAG | TTGAAATGGTCCTATGGCA | 861 |
| in-*lacZ*_1_ | GTGAAAACCGCGGCCG | TTTCCATATTTTTAGCGCCG | 620 |
| in-*lacZ*_2_ | AATGCGGGTCGCTTCACTTA | TGTGCCGAAATGGTCCATCA | 850 |
| *ilo* | CCGATGTCCTCCCTGTGAAA | ATCGGTACGCCCGGATTTTT | 730 |
| *ldh* | GCATGCTTTTAAGATGAAGTCTCAC | CAAAAAATCATTTTAGTTGGCGACGG | 1100 |
| *hly* | ATGAAAAAAATAATGCTAGTTTTTTA | TTATTCGATTCGATTGGATTATCTACT | 1590 |
| *Ery* | GTCGACGATTCACAAAAAATAGGC | ACTAGTCCCGGGGCGAATTG | 1400 |

Table S2.

**Biochemical properties of each strain**

| **Biochemical reaction** | **LM** | **LI** | **LIΔ*ilo*::*hly*** | **LIΔ*ilo*** |
| --- | --- | --- | --- | --- |
| AMY | + | + | + | + |
| ADH1 | - | - | - | - |
| BGAL | - | - | - | - |
| AGLU | + | + | + | + |
| APPA | - | - | - | - |
| AspA | - | - | - | - |
| BGAR | - | - | - | - |
| PHOS | - | - | - | - |
| LeuA | - | - | - | - |
| ProA | - | - | - | - |
| BGURr | - | - | - | - |
| AGAL | - | - | - | - |
| PyrA | - | - | - | - |
| BGUR | - | - | - | - |
| ALaA | - | - | - | - |
| dSOR | - | - | - | - |
| URE | - | - | - | - |
| dGAL | - | - | - | - |
| ILATk | - | - | - | - |
| NAG | + | + | + | + |
| dMAL | + | + | + | + |
| BACI | + | + | + | + |
| dMAN | - | - | - | - |
| dMNE | + | + | + | + |
| MBdG | + | + | + | + |
| PUL | - | - | - | - |
| dRAF | - | - | - | - |
| SAL | + | + | + | + |
| ADH2s | - | - | - | - |
| OPTO | + | + | + | + |
| PIPLC | + | - | - | - |
| DXYL | - | + | + | + |
| LAC | + | - | - | - |
| NOVO | + | - | - | + |
| NC6.5 | + | - | - | - |
| AMAN | + | - | - | - |
| SAC | + | - | - | - |
| dRIB | - | + | - | - |
| POLYB | + | + | - | + |
| O129R | + | + | - | + |
| dTRE | + | + | + | - |
| CDEX | + | + | + | - |
| TyrA | + | + | + | - |

Table S3.

**The pathology scores of mice Liver, spleen and lung after inoculation of each strain**

| **Organ tissue** | **Group** | **Time** | **Watery degeneration** | **Ballooning** | **Necrosis** | **Inflammatory cell infiltration** |
| --- | --- | --- | --- | --- | --- | --- |
| **Liver** | PBS |  | 0 | 3 | 0 | 0 |
|  | LM | 3 d | 3 | 0 | 3 | 2 |
|  |  | 14 d | 2 | 0 | 2 | 2 |
|  | LI | 3 d | 3 | 0 | 3 | 2 |
|  |  | 14 d | 0 | 0 | 0 | 2 |
|  | LIΔ*ilo*::*hly* | 3 d | 0 | 0 | 2 | 2 |
|  |  | 14 d | 0 | 0 | 0 | 2 |
|  | LIΔ*ilo* | 3 d | 2 | 0 | 0 | 2 |
|  |  | 14 d | 0 | 0 | 0 | 2 |
| **Spleen** | PBS |  | 2 | 0 | 0 | 0 |
|  | LM | 3 d | 0 | 4 | 0 | 2 |
|  |  | 14 d | 2 | 0 | 0 | 2 |
|  | LI | 3 d | 0 | 4 | 3 | 0 |
|  |  | 14 d | 0 | 0 | 0 | 0 |
|  | LIΔ*ilo*::*hly* | 3 d | 0 | 0 | 0 | 0 |
|  |  | 14 d | 0 | 0 | 0 | 0 |
|  | LIΔ*ilo* | 3 d | 0 | 0 | 0 | 0 |
|  |  | 14 d | 0 | 0 | 0 | 0 |
|  | **Group** | **Time** | **Alveolar wall thickening** | **Inflammatory cell infiltration** | **Bleeding** | **Congestion** |
| **Lung** | PBS |  | 2 | 2 | 0 | 0 |
|  | LM | 3 d | 1 | 1 | 2 | 1 |
|  |  | 14 d | 2 | 2 | 2 | 0 |
|  | LI | 3 d | 0 | 0 | 0 | 0 |
|  |  | 14 d | 1 | 1 | 0 | 2 |
|  | LIΔ*ilo*::*hly* | 3 d | 2 | 1 | 0 | 0 |
|  |  | 14 d | 2 | 2 | 0 | 0 |
|  | LIΔ*ilo* | 3 d | 1 | 1 | 0 | 0 |
|  |  | 14 d | 1 | 1 | 0 | 0 |

Note: ‘0’ means within the normal range; ‘1’ means very slight, the change just exceeds the normal range; ‘2’ means slight, lesions could be observed, but not serious, ‘3’ means moderate, the lesion is obvious and likely to be more severe; ‘4’ mean severe, the lesion is very severe (the lesion has taken up the entire tissue and organ.

Table S4.

**The approaches of determination of LD50 of each strain**

| **Inoculated bacteria** | **Vaccination route** | **Inoculation volume (μl/mice)** | **Inoculation dose (cfu)** |
| --- | --- | --- | --- |
| LM | intravenous injection | 100 | 2×10^3^ |
|  |  |  | 2×10^4^ |
|  |  |  | 2×10^5^ |
| LIΔ*ilo*::*hly* | intravenous injection | 100 | 5×10^4^ |
|  |  |  | 5×10^5^ |
|  |  |  | 5×10^6^ |
|  |  |  | 5×10^7^ |
| LIΔ*ilo* | intravenous injection | 100 | 5×10^5^ |
|  |  |  | 5×10^6^ |
|  |  |  | 5×10^7^ |
|  |  |  | 5×10^8^ |
